# MiRNA: Biological Regulator in Host-Parasite Interaction during Malaria Infection

**DOI:** 10.3390/ijerph19042395

**Published:** 2022-02-19

**Authors:** Poonam Kataria, Neha Surela, Amrendra Chaudhary, Jyoti Das

**Affiliations:** 1 Parasite-Host Biology, National Institute of Malaria Research, Dwarka, New Delhi 110077, India; punamkataria90@gmail.com (P.K.); neha.surela@gmail.com (N.S.); chaudharyamr@gmail.com (A.C.); 2 Academy of Scientific and Innovative Research (AcSIR), Ghaziabad 201002, India

**Keywords:** MicroRNAs, gene expression, biomarkers, extracellular vesicles, MicroRNAs biogenesis

## Abstract

Malaria is a severe life-threatening disease caused by the bites of parasite-infected female *Anopheles* mosquitoes. It remains a significant problem for the most vulnerable children and women. Recent research has helped establish the relationship between microRNAs (miRNAs) and many other diseases. MiRNAs are the class of small non-coding RNAs consisting of 18–23 nucleotides in length that are evolutionarily conserved and regulate gene expression at a post-transcriptional level and play a significant role in various molecular mechanisms such as cell survival, cell proliferation, and differentiation. MiRNAs can help detect malaria infection as the malaria parasite could alter the miRNA expression of the host. These alterations can be diagnosed by the molecular diagnostic tool that can indicate disease. We summarize the current understanding of miRNA during malaria infection. miRNAs can also be used as biomarkers, and initial research has unearthed their potential in diagnosing and managing various diseases such as malaria.

## 1. Introduction

Malaria is a severe vector-borne illness caused by *Plasmodium,* a protozoan parasite. The bite of infected female *Anopheles* mosquitoes facilitates the transition of the parasite to humans and other animals. According to the 2019 WHO report, 229 million malaria cases and an estimated 409,000 deaths occurred worldwide. The most susceptible groups affected by malaria are children under five and pregnant women. Malaria is caused by five species of the protozoan parasite, namely, *Plasmodium falciparum*, *Plasmodium ovale*, *Plasmodium vivax*, *Plasmodium malariae* and newly discovered *Plasmodium knowlesi.* Over 90 per cent of malaria infections are caused by *P. falciparum* compared to the other species. The Apicomplexa parasite like *Plasmodium* involves two different hosts in their life cycle, including female *Anopheles* mosquitoes as vectors (sexual cycle) and vertebrates as the final host (asexual cycle). Malaria infection begins with the injection of sporozoites into the skin of the vertebrate host from malaria-infected female *Anopheles* mosquitoes. Within 30–60 min, these sporozoites migrate through the blood to the liver and infect the hepatic cells, during which asexual multiplication occurs. These sporozoites are maturing subsequently into schizonts over 6–15 days. Eventually, each schizont multiplies to thousands of merozoites released into the bloodstream. These merozoites target specific proteins on the surface of erythrocytes and invade the cells. Through this stage, the merozoites develop to form ring stage or mature trophozoites, and then mature trophozoites develop into schizonts. This results in four to 36 new parasites in each infected cell in a 44 to72 h period. On maturation, the infected red blood cells burst, releasing the merozoites, which infect other uninfected healthy RBCs. This progression produces malarial symptoms, including chills, headache and fever and so forth. Instead of replicating, some merozoites from blood transform into gametocytes. Another mosquito then ingests these gametocytes during a blood meal. Gametocytes further develop in male and female gametes, and these gametocytes fuse inside the mosquito and form diploid zygotes, which additionally become ookinetes. These ookinetes transfer to the mosquito’s midgut and form the oocysts on the exterior wall of the midgut. Consequently, sporozoites are formed through the meiotic division of the oocysts, which further moves to the salivary glands of the female *Anopheles* and are injected into a new human host during the mosquito bite [1].

World Health Organization (WHO) has categorized malaria into severe and uncomplicated malaria. Severe malaria is caused by *P. falciparum* and is generally caused due to delay in treating an uncomplicated infection of *P. falciparum* malaria. Malaria becomes more severe when children already have comorbidities such as anemia, metabolic acidosis, organ failure, coma, and so forth. Severe malaria can be significantly controlled by the immediate treatment of malaria patients, especially in patients with an impaired immune system and a high burden of malaria parasites. The standard methods to detect malaria infection include the microscopic examination of blood films, antigen detection, and molecular testing [2]. Malaria parasite causes a headache, high fever, vomiting, extreme tiredness, clogging and ruptured blood vessels. These signs are typically visible with a delay of 15 days of infection, and the currently available molecular tools are unsuccessful in early infection. Recent research has helped establish the relationship between miRNA and many other pathogenic diseases using microarray analysis and deep sequencing [3]. Alternatively, miRNAs can help in the detection of malaria infection as in humans, malaria parasite could potentially change the expression levels of host miRNAs, and these altered miRNAs can be analyzed by the molecular diagnostic technique like microarray profiling, next-generation sequencing and quantitative reverse transcriptase-polymerase chain reaction (RT-qPCR) that can indicate infection. The alteration of miRNAs during pathological and physiological conditions has been the area of attention in the latest research. However, their role in human parasitic infections is yet to be fully explored and established.

## 2. MicroRNAs

The protein-coding genes represent approximately 2% of the entire genome sequence, and 98% of the human genome is actively transcribed in the form of non-coding RNAs, including tRNAs, rRNAs, small RNAs such as miRNAs, siRNAs and so forth [4]. MicroRNAs (miRNAs) comprise endogenous, small single-stranded natural occurring, non-coding RNAs up to 18–23 nucleotides long. MiRNAs are situated primarily on the non-coding region of the genome and play a significant role in regulating gene expression in many biological processes at the post-transcriptional level.The first miRNALin-4 was discovered in 1993 in nematodes during the genetic screening of the organism *Caenorhabditis elegans* [5]. Later, in 2000, another miRNA (let-7) was found in *C. elegans*, targeting lin-41 in many species. Lin-41 proteins are considered significant regulators of cell differentiation and proliferation. The involvement of Lin-41 appeared in both transcriptional silencing of mRNAs and ubiquitylation of protein [6]. Meanwhile, prominent miRNAs have been recognized in mammals [7]. These are evolutionally conserved in all eukaryotes and also found in some viruses. MicroRNAs play a significant role during various developments, physiological and pathological changes and in different cellular functions such as cell development, proliferation, differentiation and apoptosis.

## 3. Biogenesis of MiRNAs

**Figure 1:**
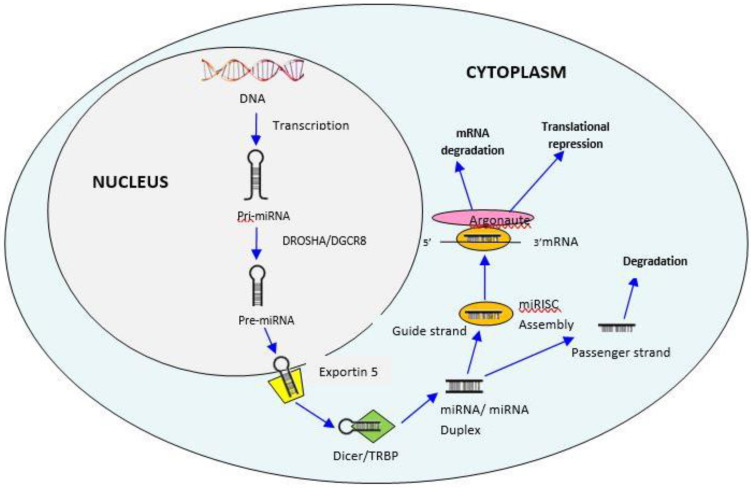
Pathway of biogenesis of microRNAs (miRNAs). In the nucleus, the non-coding part is transcribed by RNA polymerase II to form a hairpin-like structure called pri-miRNA. Then, enzymes Drosha and DGCR8 complex cleaved the pri-miRNA into pre-miRNA. From the nucleus, the pre-miRNA is exported by exportin 5 into the cytoplasm, and the complex of enzyme Dicer and Trans-Activation Responsive RNA-Binding Protein (TRBP) cuts the hairpin-like structure and processes the pre-miRNA into two unstable mature miRNA arms into two strands, mature miRNA guide strand and the miRNA passenger strand. After strand separation, the guide strand (5′end) of the miRNA/miRNA duplex is complexed with RISC attached with Argonaute protein. The miRNA RISC complex facilitates base-pairing interaction between miRNA and mRNA, which leads to gene regulation.

The biogenesis of miRNAs is a process for producing mature miRNAs, which takes place in the cell nucleus and cytoplasm. The process initiates in the nucleus, where the non-coding part of the host genome is transcribed by RNA polymerase II to produce a long hairpin-like structure of primary miRNA (pri-miRNA) having more than 200 nucleotides in length. Then, RNase III family enzymes Drosha and DGCR8 complex (DiGeorge Syndrome Critical Region 8) cropped the pri-miRNA into precursor miRNA (pre-miRNA) consisting of approximately 70 nucleotides [8]. Next, the pre-miRNA hairpin loop is transported into the cytoplasm by exportin-5. In the cytoplasm, the complex of Dicer and Trans-Activation Responsive RNA-Binding Protein (TRBP) cut the hairpin-like structure and processes the pre-miRNA into unstable 14–21 nucleotide long duplex miRNA structure and separated into two strands, mature miRNA guide strand and the miRNA passenger strand. After strand separation, the guide strand (5′end) of the miRNA/miRNA duplexis combined with the RNA induced silencing complex (RISC) attached with Argonaute protein which then interacts with the 3′ un-translated region (3′UTR) target site of the mRNA leads to the translational repression and mRNA degradation. In contrast, the passenger strand is often degraded. As miRNAs can affect multiple RNAs, a single miRNA can potentially impact numerous regulatory functions [9] (Figure 1) [10].

MiRNAs have a primary role in regulating gene expression in the human body by partially binding to mRNA, usually within the 3′ untranslated region and thus preventing their translation to protein [7]. Interestingly, a single miRNA can bind to many mRNAs and can regulate the expression of a large number of genes, and any mRNA can be regulated by several miRNAs simultaneously [11]. Moreover, miRNAs recognize the target mRNA accurately and subsequently down-regulate the gene expression either by translational repression or mRNA cleavage [7]. The backbone of the miRNA-mediated gene silencing primarily depends on the degree of complementarities between miRNA and mRNA. The high degree of complementarity between miRNA and target mRNA enables the AGO complex protein to break the target mRNA, which fixes the process of gene silencing. The Argonaute(AGO) protein complex has four isoforms, out of which only AGO 2 (known as slicer) can break the target Mrna [12]. The low degree of complementarity between miRNA and mRNA promotes the translational repression mechanism by binding with the target mRNA.

MiRNAs can be tissue-specific or species-specific. Such miRNA-122 and miRNA-124 is highly distributed in the liver and neurological tissues, respectively [13]. Researchers have revealed that miR-1found in humans; flies and worms are tissue-specific. This miRNA has been evolutionally conserved and has played an important role in muscles. On the other hand, there are many miRNAs, even though they are conserved and have hundreds of non-conserved miRNA genes. These non-conserved miRNAs might be playing a critical species-specific function [14]. While many studies have hinted the vital role of miRNAs in the pathogenesis of numerous human parasitic or bacterial or viral infections, the specific functions of miRNAs in these infections are still unclear.

## 4. MiRNAs during Malaria Infection

The malaria parasites have a complex life cycle, including sexual and asexual stages in female *Anopheles* mosquitoes and humans. At both transcriptional and post-transcriptional levels, this complex life cycle progress requires tight regulation of gene expression [12]. The asexual erythrocyte stage, including intraerythrocytic development, involves significant interaction between host erythrocyte cells and parasites. Some material is exchanged between host erythrocytes and parasites during these interactions. Curiously, this interaction leads to the transfer of human miRNA from erythrocytes into the parasite. In humans, *plasmodium* parasites invade into erythrocytes (RBCs) using cytoadherence ligand of *P. Falciparum* Erythrocyte Membrane Protein-1 (PfEMP-1 until the mature parasite sequestration [15]. Approximately200 human miRNAs are found in RBCs lacking nucleus and transcription/translation machinery [15]. Some chimeric fusions are formed between *Plasmodium falciparum* mRNA and human miRNAs, which are transferred into the *Plasmodium*, resulting in translational inhibition. Sequencing and bioinformatics searches revealed that the *P. Falciparum* genome lacks the main enzymes, such as Dicer and AGO, required for RNA interference that induce sequence-specific gene silencing by double-stranded RNA. Indeed, parasite *Plasmodium falciparum* imports these necessary enzymes along with miRNAs, including let 7a and miR-15a from the human RBC to regulate its gene expression, for example, target the *Plasmodium* gene Rad54 [16]. Moreover, the cyclic AMP signaling pathway plays a significant role in parasite development by activating molecules such as the cAMP-dependent protein kinase (PKA). In *P. falciparum*, two subunits of PKA, namely, cAMP-dependent catalytic (PKA-C) and regulatory (PKA-R), have been recognized, and the regulation of PKA-R levels is critical for *plasmodium* survival. MiR-451 integration into *P. falciparum* AMP-dependent protein kinase (PKA-R) transcript reduced the translation of regulatory PKA activity and reduced the parasite growth [17,18].

Reports revealed that high levels of specific human miR-451, miR-223 and let-7i are found in both HbAS (having sickle cell trait) and HbSS (having sickle cell disease) erythrocytes compared with normal red blood cells and negatively regulate the malaria infection [19]. Rathjen et al. (2006) have performed small RNA expression profiling from parasite-infected RBCs and healthy RBCs and found a high amount of human miRNA, most abundantly miRNA 451, both in infected RBCs and unparasitized RBCs. Therefore, miR-451 could be functional in the differentiation of erythroid cells [20]. Despite this, some miRNA like miR-451, miR-106, miR-16, miR-92, miR-7b, miR-144, miR-142, let-7f, let-7a and miR-91 are down-regulated, whereas miR-223 and miR-19b are upregulated in red blood cells of malaria parasite-infected patients. MiR-92 and miR-17 regulate the TGF-β signalling pathway related to malaria [21,22]. One more study revealed the role of two erythrocytic miRNAs mimic, miRNA-150-3p and miR-107-5p, which retarded malaria parasite growth when highly present in erythrocytes [23]. Malaria parasite released tissue-specific miRNAs into the bloodstream, and dysregulation of miRNAs such as miR-16, miR-150, miR-155, miR-223 and miR-451 are associated with immune-related genes expression including, AGO-1, AGO-2, CD36, IL4, IFN-CD80, CD86, PfEMP-1, ANG-1 and ANG-2 during malaria infections.

**Figure 2:**
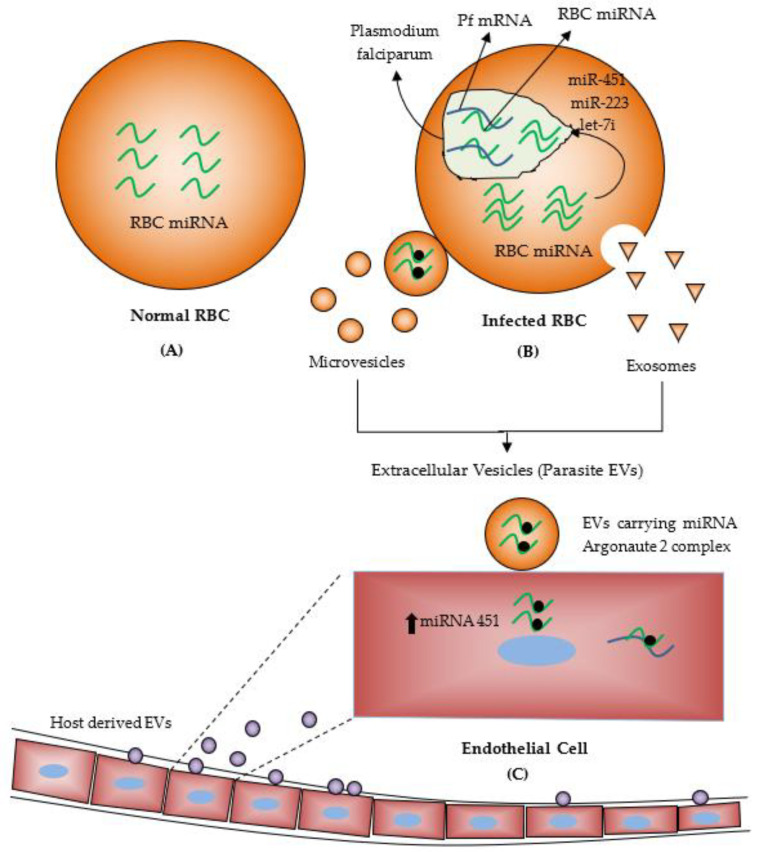
(**A**) About 200 miRNAs are found in human RBCs lacking nucleus and transcription/translation machinery. (**B**) *Plasmodium*-infected human red blood cells (RBCs). Transfer of miRNAs from RBC to *Plasmodium* parasite and inhibit translation. (**C**) Transfer of miRNAs from *Plasmodium*-infected RBC to endothelial cells. (Reproduced from Bayer-Santos, E.; Marini, M. M.; da Silveira, J. F., Non-coding RNAs in host–pathogen interactions: subversion of mammalian cell functions by protozoan parasites. Frontiers in microbiology 2017, used under CC BY 4.0 with modification and addition of Normal RBCs, Microvesicles, Exosomes and Host derived EVs [30]).

MiRNAs are significantly involved in several biological processes by regulation of gene expression. These are secreted in biological fluids like plasma, serum, cerebrospinal fluid, milk, urine, saliva, seminal fluid, tears and blood [8]. These are called circulating or extracellular miRNAs. Plasma and serum miRNAs are found in different extracellular vesicles fluids such as exosomes, microvesicles, and apoptotic bodies based on their size and cellular origin. Extracellular vesicles are small membrane-bound particles released from many types of cells. Extracellular vesicles derived miRNA, including plasma miRNA, are highly stable and consist of proteins, lipids and nucleic acids [24]. Many studies revealed that elevated release of extracellular vesicles from various cell types, including endothelial cells, RBCs and platelets, containing small RNAs, correlates with malaria severity [25,26]. For example, the elevated level of extracellular vesicles derived miRNA-15-5p and miRNA-150b-5p were found in *Plasmodium vivax*-infected patients while let-7a-5p was upregulated in both *Pv* and *pf* infected patients in Thailand [27]. A recent study explored thatMiR-451 and miR16 are the most abundant miRNAs in normal plasma and normal RBCs [24]. In contrast to another study, a significant decrease in the level of miRNA-16 and miRNA-145 in plasma of *Plasmodium vivax* infected patients compared to normal individuals but no significant difference for *pf* infected patients with normal individuals [3,28]. Therefore, a diminished level of miRNA-145 leads to lower growth of the *Plasmodium* parasite in in-vitro [29]. EVs released from infected RBCs carry functional miRNAs with Argonaute 2 complex protein in the bloodstream, and these EVs could be absorbed by the host endothelial cells. EVs delivered miRNAs alter the expression level of the target proteins and affect endothelial cell functions (Figure 2) [30]. Another study, Wang and colleagues suggested that more amounts of microparticles (extracellular vesicles) were released from a culture medium of malaria-infected RBCs than the normal RBCs. Human AGO 2 protein was found to bind with hundreds of miRNAs in these microparticlesand this miRNA-hAGO2, which is a component of the miRISC complex were transferred into the *Plasmodium falciparum* [25]. AGO proteins (Argonaute proteins) are usually associated with small RNAs and are involved in post-transcriptional gene-silencing processes. When bound to miRNAs, AGO proteins also stabilize and protect miRNAs from degradation in animals and plants. Many studies also reported that EVs miRNAs could be transferred from one species to another and play crucial roles in cell-to-cell communication [31].

Several studies were also performed on an animal model for indicating miRNA expression in the malaria model. Cerebral malaria (CM) is a fatal complication of *Plasmodium* infection, mostly affecting children. MiRNAs play an important role in regulating immune cell responses against infection. The infection of *Plasmodium chabaudi* and *P.ANKA* in the mice experiment model showed changes in the miRNA expression and caused the development of protective immunity against the *Plasmodium* parasite. The up-regulation ofmiR-27a, miR-150 and let7i was seen in the brain of *Plasmodium berghei* infected CBA mice that cause cerebral malaria when compared to non-cerebral malaria (NCB) and non-infected (NI) samples [32]. Another study also analyzed the change in the expression profile of some miRNA by using the microarrays technique in microvesicles from *Plasmodium* *ANKA* and *P.yoelii* infected CBA mice that cause cerebral malaria and severe malaria and found miR-146a and miR-193b were rich in microvesicles from cerebral malaria-infected mice when compared with non-cerebral malaria and non-infected mice [33]. MiRNAs may regulate biological pathways related to innate and adaptive immunity. Other studies analyzed dysregulated twelve, miRNAs. (MiR-21-5p,-18a-5p,-19a-3p, -20b-5p, -142-3p, -27a-5p, -152-3p, -193a-5p, -155-5p,-218-1-3p, -543, and -411-5p) between non-infected (NI) and cerebral malaria (CM). Three miRNAs, miR-142-3p, miR-27a-5p andmiR-19a-3p, were considerably upregulated in CM mice compared to NI and NCM. These miRNAs are significantly involved in some cerebral malaria-like adherens junctions, FoxO, TGF-β and endocytosis pathways [34]. MiRNA-146a plays an important role in innate immunity responses, including IL-receptor-associated kinase (IRAK)-1 and TNF receptor-associated factor (TRAF)-6 (Table 1) [35].

## 5. MiRNAs as Diagnostic Tools for Malaria and Other Diseases

Diagnostic methods for malaria include microscopy, rapid diagnostic test (RDT), molecular diagnosis including polymerase chain reaction (PCR), quantitative PCR (qPCR) or reverse transcriptase PCR(RT-PCR), semi-nested PCR (n-PCR), multiplex PCR(m-PCR), indirect fluorescent antibody test and isothermal methods, viz, loop-mediated isothermal amplification (RPA) etc. [36]. While microscopy is the “Gold Standard” method for the detection of malaria in endemic regions, in the absence of well-trained technicians, it has low sensitivity and is time consuming compared to other methods. Rapid diagnostic tests (RDTs) are the primary tool for malaria diagnosis. Still, it is based on the detection of histidine-rich protein II (HRP2) produced by the parasites during the erythrocytic stage of the infection. However, RDTs can yield false positives as HRP2 remains in the blood for several days after the clearance of infection. Also, the first reports of false-negative mRDTs results due to pfhrp2 and pfhrp3 deletions in the Amazon region of Peru and Eritrea has emerged as new complexity in HRP2 related applications [37]. However, the most sensitive method for malaria diagnosis is the Polymerase chain reaction (PCR) which detects parasitemia as low as 2–5 parasites/μL. Still, it is complex and expensive [38]. Nested PCR is more effective than RDTs and microscopy, but it is time consuming and costly. Multiplex PCR can be used to overcome the shortcomings of Nested PCR as it amplifies more than one target at a time while using fewer chemicals and consumables. Still, it used target-specific primers and defined protocols. qPCR also needs sophisticated machines and well-trained technicians to conduct the test and interpret results, thus making it unsuitable for routine malaria diagnosis. Another method for malaria diagnosis is Immunofluorescence antibody testing (IFA), a sensitive and straightforward serologic test for malaria detection. Still, it is time-consuming and also requires fluorescence microscopy and trained technicians [2]. Other developed techniques for malaria diagnostic, including loop-mediated isothermal amplification(LAMP), RNA hybridization assays, DNA amplification method, laser desorption mass spectrometry(LDMS) and so forth, still have limitations to detect some parasite stages [36].

Given the limitations of the current malaria diagnostic tools, miRNAs, which are non-coding RNA molecules present in tissues, most cell types and all body fluids, have emerged as an up-and-coming diagnostic tool for infectious diseases [39]. MiRNAs represent a trustworthy indicator of infection even before the appearance of clinical symptoms as they are sensitive to pathological and physiological variation in blood or plasma [40]. Moreover, high stability against temperature and pH, resistance to ribonucleases and physicochemical conditions in body fluids increase the feasibility of using miRNAs as biomarkers for clinical applications [41]. Also, miRNA molecules preserved with formalin-fixed paraffin-embedding (FFPE) processing are more stable, economic than frozen samples and have preserved histological properties of the sample. An excellent correlation indeep sequencing of FFPE samples with matched frozen samples has been reported in several reports [42,43]. During infection, the variation in the composition and levels of extracellular miRNAs in the blood or plasma make them potential biomarkers in diagnosing infectious diseases [44,45]. Several studies have assessed the potential of miRNAs as the biomarkers of numerous diseases and their ability to improve disease outcomes [39]. Firstly, Lawrie et al. [41] reported miRNAsas a biomarker for cancer in 2008. They were utilized miRNAs to evaluate disperse large B-cell lymphoma in the serum of the patients [41,46]. Alteration in miRNAs profile of rabies infected brain tissue were reported [47]. Several miRNAs (miR-187, miR-100, miR-706, miR-322, miR-98, and miR-466) differentially expressed during varicella zoster infection in the serum of infected mice and these miRNAs have immense potential for the diagnosis of varicella zoster infection [48]. Recently, four miRNAs (miR-195-5p, miR-223-3p, miR-16-5p, and miR-20b-5p) were tested as valuable biomarkers of both HIV infection and AIDS-related diseases diagnostics [49]. The expression level of miRNA-22 is upregulated in serum of patients with coronary slow flow (CSF) as compared to the normal subjects and may serve as a biomarker for early diagnosis of CSF [50]. Moreover, miRNAs have shown great potential as diagnostic biomarkers for neurodegenerative disorders [51], cardiovascular diseases [52], endometriosis [53]; cancer [46] and several other bacterial, viral and parasitic diseases (Table 2).

## 6. Conclusions

The research on microRNAs has evolved; the significance of small non-coding RNA molecules in malaria and other diseases has been well documented. The advent of new techniques, such as deep sequencing and microarray analysis, has revealed the altered expression patterns of the specific host miRNAs during the virus, bacteria and other pathogenic infections. MiRNAsare also significantly involved in intercellular communication and regulate host cell activity, which plays a vital role in the host immune response to the malaria parasite. Moreover, miRNAs expression assessment in body fluids likes plasma, serum and so forth can be used as a promising biomarker for detecting malaria. This review has tried to summarize the changes reported in host miRNAs and their response after malaria infection based on current literature. Other miRNAs research on malaria infection may help as a malaria diagnostic tool in the early detection of severe malaria and its prognosis.

## Figures and Tables

**Table 1:** List of miRNAs studies conducted on humans and animal model experiments to explore various miRNA regulations and their functions.

S. No	Study/Author	Year	MiRNA	Regulation	Functions
1.	Ketprasit et al. [27]	2020	MiR-150-5p andMiR-15b-5p	Upregulated level of extracellular derived miRNA in *P. vivax* infected patient	Adherens junction and TGF-beta signalling pathway.
			let-7a-5p	Upregulated in both *P. vivax*- and *P. falciparum* infected patients	Adherens junction
2.	Aarón Martin-Alonso et al. [34]	2018	miR-19a-3p, miR-27a-5p, and miR-142-3p	It is upregulated in CM infected mice’s brains compared to NI and NCM.	Play a significant role in several pathways relevant to CM, including the TGF-β and endocytosis pathways.
3.	Cohen et al. [33]	2018	miR-146a and miR-193b	Upregulated in microvesicles from cerebral malaria-infected mice	Cerebral pathology
4.	Chamnanchanunt et al. [3]	2015	MiR-145 and miR-16	Down-regulated in serum of *P. vivax* infected patients	Not defined
			miR-223, miR-226-3p	No change	Not defined
5.	LaMonte et al. [19]	2012	MiRNA-145, MiRNA-223 and let-7i	It is upregulated in HbAS and HbSS erythrocytes of *P. falciparum* infected patients.	Integrated into parasite mRNAs and resulted in translational inhibition.
6.	El-Assaad et al. [32]	2011	MiR-27a, miR-150	Upregulated in the brain tissue of PbA infected mice	Cell proliferation, development, and differentiation.
			let-7i	Upregulated in the brain tissue of PbA infected mice	Cellular proliferation and the innate immune response
7.	Rathjen et al. [20]	2006	MiR-145	Upregulated in both infected and healthy red blood cells	Differentiation of erythroid cells.

**Table 2:** List of various miRNAs altered during non-infectious, viral, bacterial, protozoan diseases.

S. No.	Disease	Type of Disease	Effect on MiRNAs	References
1.	Chronic Lymphocytic Leukemia	Non-infectious	Altered miRNA expression pattern in patients who have chronic lymphocytic leukaemia.	Calin et al., 2002 [54]
2.	Colorectal Neoplasia	Non-infectious	Murine miRNAs (miR-143 and miR-145) showed reduced steady-state concentrations at different cancer stages of colorectal neoplasia.	Michael et al., 2003 [55]
3.	Human cancers	Non-infectious	Downregulation of miRNAs in tumours compared with normal tissues was observed. The potential of miRNA profiling in cancer diagnosis was highlighted.	Lu et al., 2005 [56]
4.	Sepsis	Viral infection	miR-15a, miR-122, miR-4661, miR-483-5p, miR-342-5p miR-297, miR-181b, and miR-193 were, while miR-486, miR182, miR-4772, miR-574-5p, and miR-133a were found to be upregulated.	Ojha R et al., 2019 [44]
5.	Human Immunodeficiency Virus	Viral infection	miR-33a-5p, hsa-miR-223, hsa-miR-146a-5p, and miR-29b-3p were downregulated in the infected individuals.	Houzet et al., 2008 [57]
6.	Hepatitis	Viral infection	MiR-34, miR-4485, miR-92b-5p, miR-200b-5p, miR-29b and miR-192b-5p were upregulated, whereas miR-125, miR-330-3p, miR-1468, and miR-3180 were downregulated in infected individuals.	Xu et al., 2011 [58]
7.	Tuberculosis	Bacterial infection	miR-889, miR-576-3p and miR-361-5p were elevated in tuberculosis-infected serum	Qi et al., 2012 [48]
8.	Helicobacter pylori	Bacterial infection	The level of miR-17-p, miR-106a, miR-21, andmiR-106b were upregulated, and the expression of let-7a was downregulated.	W. K. K. Wu et al., 2010 [59]
9.	Malaria	Protozoan infection	miRNA-16, miRNA-106, miRNA-91, MiRNA-451, miRNA-144, miRNA-7b, miRNA-142, miRNA-92 let-7a, and let-7f, were downregulated, whereas miRNA-19b and miRNA-223 were upregulated in the RBCs.	LaMonte et al., 2012 [17]
10.	Trypanosomiasis	Protozoan infection	the expression level of miR-338 and miR-193b remarkably increased in patients affected with trypanosomiasis, whereas the expression of miR144 decreases at the time of infection	J. Wang, He, et al., 2012; Liu et al., 2011 [60]

## Data Availability

Not applicable.
